# The veridical Near-Death Experience Scale: construction and a first validation with human and artificial raters

**DOI:** 10.3389/fpsyg.2025.1661390

**Published:** 2025-10-16

**Authors:** Bruce Greyson, Jeffrey Long, Janice Miner Holden, Jean-Pierre Jourdan, Robert A. King, Suzanne Mays, Robert Mays, Titus Rivas, Natasha Tassell-Matamua, Pim van Lommel, Marjorie Woollacott, Patrizio Tressoldi

**Affiliations:** ^1^Division of Perceptual Studies, University of Virginia, Charlottesville, VA, United States; ^2^Near-Death Experience Research Foundation, Georgetown, KY, United States; ^3^University of North Texas, Denton, TX, United States; ^4^IANDS-France, Oraison, France; ^5^NDE OBE Research Project, Casa Grande, AZ, United States; ^6^IEEE-ORG, Chapel Hill, NC, United States; ^7^Athanasia Workgroup, Nijmegen, Netherlands; ^8^School of Psychology, Massey University, North Palmerston, New Zealand; ^9^Rijnstate Hospital, Arnhem, Netherlands; ^10^Institute of Neuroscience, University of Oregon, Eugene, OR, United States; ^11^Science of Consciousness Research Group, Studium Patavinum, Padova University, Padua, Italy

**Keywords:** near-death experiences, veridical perception, mind-brain relationship, consciousness, Large-Language Models, artificial intelligence

## Abstract

**Introduction:**

In this study, we describe the construction of the veridical Near-Death Experience Scale (vNDE Scale), a structured instrument for evaluating the evidential strength of perceptions reported during near-death experiences (NDEs), and its first validation by human and artificial raters.

**Methods:**

The construction was implemented using a typical Delphi Method. The first draft of the scale was evaluated by 13 experts in NDE, who were asked to suggest revisions and comments within a month for the first round and 20 days for the second round.

**Results:**

A general consensus was achieved on the second round on eight criteria related to the timing of the investigation, the medical and physical conditions, the level of third-person verification, and the number, type, and quality of perceptions reported by the near-death experiencer, to be rated on a four-level Likert scale. The validation phase consisted of the application of the vNDE Scale to 17 cases of potentially veridical NDEs by 11 independent human raters and three artificial raters based on Large-Language Models. In 14 of the17 cases (82.3%), the overall agreement between human and artificial judges was over 75%, considering the two close levels of evidence strength, i.e., moderate plus strong, low plus very low, or vice-versa.

**Discussion:**

The vNDE Scale is a practical tool for evaluating the evidential strength of perceptions reported by near-death experiencers.

## Introduction

### Background on near-death experiences and veridical perception

Near-death experiences (NDEs) are typically defined as episodes of disconnected consciousness occurring in the context of life-threatening conditions, such as cardiac arrest, severe trauma, perioperative complications, or asphyxia, during which individuals report vivid perceptions and memories despite apparent unresponsiveness or lack of normal sensory input (Fritz et al., 2024). Core phenomenological features often include a sense of peacefulness, out-of-body experiences (OBEs), altered time perception, encounters with bright light or otherworldly environments, and, in some cases, interactions with nonphysical beings or with deceased persons. These accounts are frequently transformative, influencing experiencers' attitudes toward life and death (Greyson, 2022; Long and Woollacott, 2024; Miquel-Sendra and Garcia-Alandete, 2025).

Within NDE research, one of the most compelling and controversial aspects is the veridical perception during the experience (vpNDE). Veridical perceptions refer to sensory or perceptual reports (visual, auditory, kinesthetic, olfactory, etc.) that individuals claim to have had during an NDE and that are subsequently corroborated by independent observers. Critically, veridical NDE perceptions sometimes occur under conditions in which normal sensory access seems impossible given the experiencer's physical state (e.g., flatlined EEG, lack of visual access; Sabom, 1998), thus appearing to challenge standard neurophysiological accounts of perception and of consciousness. Examples include reported observations of unusual surgical team behavior confirmed by attending staff (Cook et al., 1998) and reports of encountering a deceased person unknown to the experiencer at the time, but later verified to have died shortly before the NDE (Greyson, 2010). Such anomalous corroborations, when rigorously documented, have fueled debates about the nature of consciousness and its potential independence from brain activity.

### Review of veridical NDE cases and challenges in evaluation

Systematic literature reviews and case collections over the past 150 years include a multitude of reported instances of veridical perceptions during NDEs. For instance, Holden's review identified more than a hundred cases in which experiencers described accurate details of events in the physical environment that seemed inaccessible via ordinary sensory channels (Holden, 2009).

In a recent book, Rivas et al. (2023) presented over 120 cases of vpNDE which they referred to as “verified paranormal phenomena within near-death experiences.” To our knowledge, this book represents the most comprehensive collection of such cases in professional literature in which perception was not just verified by the NDErs themselves but directly confirmed by third parties. The cases were obtained from a variety of sources, both peer-reviewed and popular sources. Although in academia peer-reviewed sources are most highly valued, in Rivas et al.'s book, the authors investigated each case to confirm its veracity.

Nonetheless, rigorous scientific scrutiny remains challenging because many reported cases lack precise timing confirmation (i.e., ensuring perceptions occurred during periods of clinical death or flatlined brain activity), robust documentation protocols, or standardized criteria for evaluating credibility. Moreover, while some investigators argue that the accumulation of veridical reports strengthens the evidence for non-local consciousness, skeptics point out methodological limitations, potential retrospective biases, and difficulties in excluding subtle sensory or inferential processes from the analysis.

Empirical paradigms have been proposed, such as placing hidden visual targets in likely resuscitation settings to test for OBEs, but results have been limited by practical and ethical constraints, low occurrence rates of NDEs under controlled conditions, and difficulty in ensuring true “blind” conditions during crises (Parnia et al., 2014, 2023). Consequently, there is no universally accepted standard for classifying or scoring veridical NDE phenomena, leading to inconsistent interpretations among researchers.

### Rationale for developing a veridical NDE scale

Given the importance of veridical perceptions for theoretical debates about mind–brain relations and the current heterogeneity in case evaluation, there is a clear need for a systematic, reproducible instrument to assess reports of veridical NDE perceptions. A dedicated veridical Near-Death Experience Scale (vNDE Scale) can provide several benefits:

- Standardization: By operationalizing criteria for veridicality the scale can reduce subjective variability across evaluators.
- Reliability Assessment: It allows quantification of inter-rater agreement among human and artificial experts.
- Comparability Across Studies: Researchers can use a common scoring system to compare case sets from diverse sources (peer-reviewed, anecdotal, archival), facilitating meta-analyses.
- Incorporation of AI Raters: Recent advances in Large-Language Models (LLMs) for qualitative coding suggest the potential for artificial raters to assist or augment human evaluation of narrative reports. Validating AI against expert consensus can expand scalability when processing large corpora of NDE accounts.

### Expert consensus and item development

We adopted an expert consensus procedure, akin to the Delphi methodology, to generate and refine scale items. Initially, a comprehensive literature review identified dimensions relevant to veridical NDE perceptions: modality of perception, degree of anomaly relative to sensory access, quality and number of corroborating witnesses, timing of perception(s) (e.g., documented flatline periods), environmental conditions, and potential alternative explanations (e.g., pre-existing knowledge and inference). Preliminary item pools were drafted to reflect these dimensions. In subsequent rounds, independent panels of NDE researchers, clinicians, and methodologists rated the relevance, clarity, and representativeness of the items. Content validity metrics (e.g., content validity ratio, expert agreement indices) guided item retention and revision.

### Incorporating artificial raters

In parallel with human expert evaluation, we explored the feasibility of AI-assisted rating using state-of-the-art Large-Language Models (LLMs). Recent studies have demonstrated that LLMs can perform deductive coding with agreement levels comparable to human coders when guided by well-designed prompts and iterative validation procedures (Mens et al., 2023; Feuerriegel et al., 2025*)*.

### Aim of the study

The present study reports the construction of the vNDE Scale and first validation findings based on a pool of veridical NDE case descriptions drawn from peer-reviewed publications. Key objectives included (1) documenting content validity, (2) documenting expert consensus on criteria relevance and coverage of the veridical perception construct, (3) assessing the inter-rater reliability among human experts applying the scale to a set of cases, (4) evaluating the level of agreement between LLM-based ratings and human expert ratings, identifying the strengths and limitations of artificial and human raters, and (5) providing practical guidelines for future applications of the vNDE Scale in prospective data collection, retrospective case reviews, and large-scale qualitative analyses are discussed.

By establishing a rigorously developed and preliminarily validated vNDE Scale, we aim to provide the NDE research community with a tool to systematically assess veridical perceptions, thereby enhancing methodological rigor, facilitating comparisons across studies and informing theoretical debates on consciousness.

## Methods

### Participants

The participants were leading figures in the field of near-death studies and experts who had addressed NDEs using a scientific approach. They were recruited for both scale construction and its first validation.

Sixteen experts were selected and invited individually (including the project originators, authors BG and PT), offering co-authorship of the scale and the present paper for their collaboration. Thirteen (81.3%) experts accepted the invitation. The identity of those who accepted the invitation for the scale construction and its validation is disclosed in the authors of the scale (see Supplementary material) and as authors of this study, respectively.

### Scale construction

Scale construction followed the typical Delphi Method and the procedures suggested by Kekecs et al. (2020). Authors BG and PT sent a draft of the scale to the 11 other experts, asking them to suggest revisions and comments within a month for the first round and 20 days for the second round.

The first draft of the scale comprised seven criteria to be rated on a four-level Likert scale, with a request to explain the rating choice to obtain qualitative information.

The criteria to rate the potential vNDE comprised items related to the timing of the investigation, the medical and physical conditions, the level of third-party verification, and the number, type, and quality of perceptions reported by the near-death experiencer.

A consensus of 80% on all items was achieved after two rounds. The exact wording of the scale items is reported in the Supplementary material and consists of the following eight criteria:

- Timing of investigation: The period of time between when the NDE occurred and when investigation of veridical aspects of the NDE was initiated.
- Physical state of non-responsivity (unconsciousness): The timing and associated condition of the perception(s), indicating whether they occurred during physical non-responsivity, reported by the medical staff.
- Cardiac or respiratory arrest, or cessation of brain activity: The timing and associated condition of the perception(s), indicating whether they occurred during cardiac or respiratory arrest or cessation of brain function, reported by the medical staff.
- Third-person verification: Accuracy of the perceptions verified by at least one credible source besides the near-death experiencer, such as medical personnel or other trustworthy witnesses, documented through sources as published testimony, interview, and/or medical records.
- Possible physical explanation: The nature of the perception was such that it could be accounted for through physical sensory cues or logical inference.
- Number of verified perceptions: Number of verified persons, objects, environment characteristics or events perceived in the physical environment, whether immediate (e.g., hospital) or remote (e.g. in a distant city or house), during the period(s) of physical non-responsivity.
- Erroneous perceptions: Number of persons, objects, environment characteristics or events that the experiencer reported having perceived during the NDE yet were later found to be inaccurate perceptions.
- Clarity of verified perceptions: Clarity of the experiencer's reported verified perceptions and the memory of them.

For each criterion, raters were invited to provide a written explanation of their rating. These justifications were valuable for understanding the rationale behind each score and for identifying the specific sources or evidence informing the rater's evaluation.

### Scale scoring

The scale scoring consisted of the sum of the scores assigned to the eight items, and their transformation into four levels of evidential strength, corresponding to the quartiles of the score distribution, ranging from 0 to 32, not on frequency distributions of the ratings:

First quartile (8–14): Very Low evidential strength

Second quartile (15–20): Low evidential strength

Third quartile (21–26): Moderate evidential strength

Fourth quartile (27–32): Strong evidential strength, with a score of 3 or 4 on criterion 4.

### Scale validation

Scale validation comprised the application of the scale to 17 potential vpNDE, described in nine peer-reviewed scientific papers, by 13 experts and the free version of three AI software based on Large-Language Models, ChatGPT v.4, Gemini Pro, and Mistral Medium 3. The inclusion of AI raters served two primary purposes: a) to explore the feasibility of using LLMs for rapid and scalable application of the scale, and b) to help mitigate potential biases in human expert evaluations. For instance, concerning Criterion 5 (“Possible Physical Explanation”), an expert who holds the belief that NDEs demonstrate the capacity of consciousness to operate independently of the brain may be inclined to rate the absence of a physical explanation more favorably. The use of AI provides an additional layer of evaluation that is potentially less influenced by such interpretive biases.

The selection of the scientific papers describing potential vpNDE was made by authors BG and PT following these unique inclusion criterion: papers describing a single or multiple single cases with sufficient details published in peer-reviewed scientific journals.

The list of all the cases and the nine papers selected are available at: https://zenodo.org/records/16949744.

All 13 experts, including BG and PT, were invited to complete an online version of the vNDE Scale for each of the 17 cases described in the nine selected papers. A total of 11 responses were received: two experts opted to collaborate and submit a joint evaluation, while one expert was unable to complete the task within the designated time frame.

For the AI softwares the procedure was the following one: After having uploaded the vNDE Scale, and a copy of the paper, the prompt for the three AI software was: “*Read carefully the Veridical Near-Death Experience Scale (vNDE Scale), and apply it to the case [case identity] described in the study [title of the file] based on the information provided in the [Methods and Results sections, or others paper section where the case and its investigation is described]*.”

All the prompts used are described in the file AIPrompts and are available at: https://zenodo.org/records/16949744.

### Data availability

The evaluation of all human experts and of the three AI raters are available open access at: https://zenodo.org/records/16949744 for independent analyses.

### Data analyses

The most important outcome of the analysis is the percentage of agreement on the assigned levels of evidential strength for each of the 17 cases, as rated by both human experts and AI systems.

### Ethics declarations

All participants were invited and accepted to be co-authors of the study, and all agreed to disclose their identities. Although AI LLMs were used, in parallel with human raters, to score cases of vpNDE, no AI programs were used in the writing or editing of this paper.

## Results

### Raters' agreement

One human rater completed the vNDE Scale for only 7 of the 17 cases, while the remaining 10 human raters evaluated all 17 cases. Table 1 presents, for each case, the highest percentage of agreement on the assigned level of evidential strength among the human raters and the three AI raters.

**Table 1:** Highest percentages of levels of evidential strength agreement among human and AI raters for each case in descending order.

**Reference**	**Level of evidential strength**	**Overall (n raters)**	**Human (n raters)**	**AI (n raters)**
Rivas and Smit (2013)	Strong	**78.6** (11/14)	**72.8** (8/11)	**100** (3/3)
	Moderate	14.3 (2/14)	18.2 (2/11)	
Parnia et al. (2014)	Strong	**71.4** (10/14)	**63.6** (7/11)	**100** (3/3)
	Moderate	28.6 (4/14)	36.4 (4/11)	
Beauregard et al. (2012)	Moderate	**71.4** (10/14)	**72.8** (8/11)	**66.6** (2/3)
	Strong	14.3 (2/14)	9 (1/11)	33.3 (1/3)
	Low	14.3 (2/14)	18.2 (2/11)	
Cook et al. (1998) – case Jennifer Edwards	Very Low	69.2 (9/13)	80 (8/10)	33.3 (1/3)
	Low	31 (4/13)	20 (2/10)	66.6 (2/3)
Cook et al. (1998) – case Al Sullivan	Moderate	69.2 (9/13)	80 (8/10)	33.3 (1/3)
	Strong	15.4 (2/13)	–	66.6 (2/3)
Woollacott (2024)	Strong	64.3 (9/14)	54.5 (6/11)	**100** (3/3)
	Moderate	28.6 (4/14)	36.3 (4/11)	
Smit (2008)	Strong	64.3 (9/14)	54.5 (6/11)	**100** (3/3)
	Moderate	28.6 (4/14)	36.3 (4/11)	
Ring and Lawrence (1993) – case Three	Moderate	61.5 (8/13)	60 (6/10)	**66.6** (2/3)
	Strong	31 (4/13)	30 (3/10)	33.3 (1/3)
Cook et al. (1998) – case Rose Heathe	Very Low	61.5 (8/13)	80 (8/10)	–
	Low	31 (4/13)	20 (2/10)	66.6 (2/3)
Ring and Lawrence (1993) – case Three	Moderate	61.5 (8/13)	60 (6/10)	**66.6** (2/3)
	Strong	31 (4/13)	30 (3/10)	33.3 (1/3)
Ring and Lawrence (1993) – case One	Moderate	61.5 (8/13)	60 (6/10)	**66.6** (2/3)
	Strong	15.4 (2/13)	10 (1/10)	33.3 (1/3)
	Low	15.4 (2/13)	20 (2/10)	–
Cook et al. (1998) – case Jean Morrow	Low	53.8 (7/13)	50 (5/10)	**66.6** (2/3)
	Very Low	31 (4/13)	40 (4/10)	–
Woollacott and Peyton (2021)	Moderate	50 (7/14)	63.6 (7/11)	–
	Strong	42.8 (6/14)	27.2 (3/11)	100 (3/3)
Sartori et al. (2006)	Moderate	50 (7/14)	54.5 (6/11)	33.3 (1/3)
	Strong	50 (7/14)	45.4 (5/11)	66.6 (2/3)
Ring and Lawrence (1993) – case Two	Moderate	46.1 (6/13)	40 (4/10)	**66.6** (2/3)
	Low	38.5 (5/13)	50 (5/10)	–
Cook et al. (1998) – case Linda McKnight	Low	46.1 (6/13)	60 (6/10)	–
	Very Low	23 (3/13)	30 (3/10)	–
	Moderate	23 (3/13)	10 (1/10)	66.6 (2/3)
Cook et al. (1998) – case Peggy Raso	Moderate	46.1 (6/13)	50 (5/10)	33.3 (1/3)
	Strong	31 (4/13)	30 (3/10)	33.3 (1/3)
Cook et al. (1998) – case Stefan von Jankovich	Low	38.5 (5/13)	50 (5/10)	–
	Moderate	31 (4/13)	30 (3/10)	33.3 (1/3)
	Strong	(4/13)	20 (2/10)	66.6 (2/3)

Only three cases achieved over 70% agreement among raters for a single, specific level of evidential strength. However, when adjacent levels are combined, such as Moderate plus Strong or Low plus Very Low, 14 out of the 17 cases (82.3%) reached a consensus level exceeding 75%. Bold percentages values correspond to percentages when at least two AI raters agreed with the highest level of evidential strength identified by the majority of human raters.

**Figure 1:**
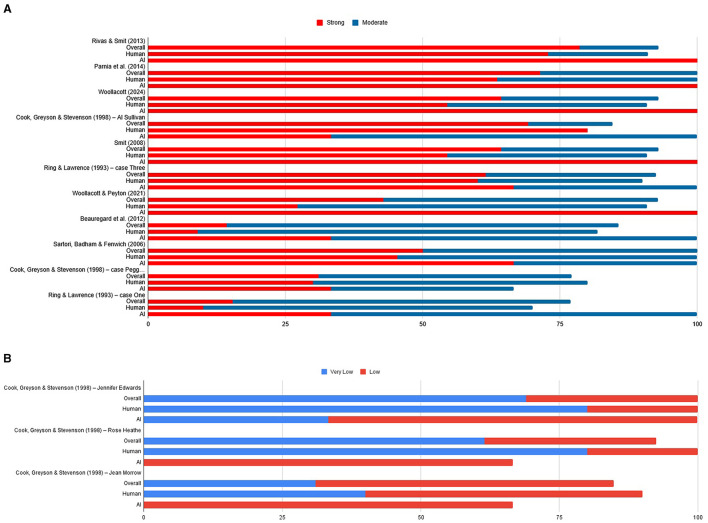
**(A)** Stacked percentages of overall agreement on the Strong and Moderate evidential strength above 75% related to the 17 cases. **(B)** Stacked percentages of overall agreement on the Very Low and Low evidential strength above 75% related to the 17 cases.

These results are presented in the following Figures 1A, B.

### AI raters

Table 2 presents the number and percentage of cases in which the three AI raters agreed on the assigned level of evidential strength. Only one case—Peggy Raso, as described in Cook et al. (1998)—was assigned different levels of evidential strength by all three AI raters. The majority of cases (64.7%) were classified at the same evidential strength level by two of the three AI raters, while the remaining 29.4% of cases received identical classifications from all three AI systems.

**Table 2:** Number and the percentage of agreement on the levels of evidential strength among the three AI raters.

**Agreement**	**Agreement percentages (*n* cases)**
3 out 3	29.4 (5)
2 out 3	64.7 (11)

### Agreement between human and AI raters

For 10 out of the 17 cases (58.8%), at least two AI raters agreed with the highest level of evidential strength identified by the majority of human raters (see bolded percentages). When adjacent levels of evidential strength are combined—such as Moderate plus Strong or Very Low plus Low—this agreement increases to 82.3% (14 out of 17 cases).

A measure of the variability of the judges scores assigned to the eight items of the scale can be obtained by examining their standard deviations. For the human judges, the standard deviations ranged from a maximum of 1.34 for item 7, “Erroneous perceptions,” to a minimum of 0.9 for item 2, “Physical state of non-responsivity.”

For the artificial judges, the standard deviations ranged from a maximum of 1.35 for item 1, “Timing of investigation,” to a minimum of 0.48 for item 5, “Possible physical explanation.”

### Krippendorff's alpha test

Overall quantitative inter-rater agreement was assessed using Krippendorff's alpha, a nonparametric statistic that estimates the consistency of agreement among multiple raters across various data types, including nominal, ordinal, interval, and ratio scales. The analysis was conducted using the Krippendorffsalpha package (Hughes, 2021).

Krippendorff's alpha ranges from 0 to 1, analogous to a correlation coefficient, with higher values indicating greater agreement. The overall alpha across all human and AI raters was 0.49 (95% CI: 0.40–0.58), indicating moderate agreement. No substantial differences in agreement were observed between human and AI raters. This result provides a useful benchmark for future validations of the vNDE Scale.

## Discussion

This study aimed to develop a scale for evaluating the veracity of perceptions reported during near-death experiences (NDEs) and to conduct an initial inter-rater validation of the instrument. The vNDE Scale was constructed through a consensus-driven process involving thirteen researchers with recognized expertise in empirical NDE investigations.

Inter-rater validation was conducted using ratings from eleven human experts and three AI software based on LLMs. When considering agreement across two adjacent levels of evidential strength (i.e., Strong plus Moderate or Low plus Very Low), consensus was achieved in 14 out of 17 cases (82.3%), suggesting a promising level of agreement for a first validation.

However, if agreement is assessed based solely on a single, discrete level of evidential strength (e.g., only Strong or only Moderate), only three cases surpassed the 70% consensus threshold, indicating more limited inter-rater reliability under stricter criteria. Thus, while consensus at a single level remains modest, the broader two-tiered agreement provides preliminary support for the scale's reliability.

An important consideration in interpreting these results is the variability in the quantity and quality of information available for each case. Discrepancies in ratings may stem not only from differences in interpretation among raters but also from disparities in the richness of case descriptions. For example, the second case described in Ring and Lawrence (1993) consists of just 155 words, whereas the case reported by Woollacott (2024) spans approximately five pages. In the latter instance, raters had access to significantly more detail, potentially leading to more informed and consistent evaluations. This variability highlights the need for standardized case reporting practices in future applications of the vNDE Scale.

### Study limitations

The primary limitation of this study is that validation was conducted using only 17 selected cases. Additional validation with a larger and more diverse set of cases will be essential to further assess and strengthen the reliability and generalizability of the vNDE Scale.

### Scale use recommendations

As an initial control step, users should evaluate Criterion 4: “Third-Person Verification.” If this criterion is rated as 1 or 2, application of the vNDE Scale should be discontinued, as insufficient third-party corroboration undermines the evidentiary value of all subsequent ratings.

Furthermore, if criterion 7, “Erroneous perceptions” is rated as 1 (all erroneous perceptions), end the scale application given that without a minimum of verified perceptions, the case should not be considered as a vNDE.

For those interested in incorporating AI raters to enhance the reliability and scalability of evaluations, it is recommended to apply the scale using at least two AI models. Rating explanations generated by the AI should be cross-checked against the original source material to ensure accuracy and interpretive validity.

The integration of human and AI raters represents a novel hybrid approach to qualitative evaluation, combining the efficiency and scalability of artificial intelligence with the contextual discernment of expert reviewers. Future research involving larger and more diverse case sets may further refine AI prompting strategies and support the development of predictive or explanatory models linking vNDE Scale scores to contextual and experiential variables.

## Conclusion

Veridical perception in near-death experiences is a fascinating phenomenon that challenges our understanding of consciousness and its relationship to the physical body. This aspect of NDEs involves perceptions reported by experiencers that are later corroborated as corresponding to material reality, despite the apparent impossibility of obtaining such information through normal sensory processes.

Within the field of near-death studies, vpNDE holds particular importance. It lends empirical credibility to experiencers' claims regarding the reality of their perceptions and supports the possibility that consciousness can persist independently of the physical body. Nevertheless, significant methodological challenges remain, including the difficulty of documenting vpNDEs under controlled conditions and the absence of a standardized framework for their evaluation. The vNDE Scale introduced in this study represents a first step toward addressing these challenges by providing a systematic tool for assessing veridical perception in NDEs. Ongoing application and refinement of the scale will be essential for improving its reliability, validity, and utility in future research.

## Data Availability

The datasets presented in this study can be found in online repositories. The names of the repository/repositories and accession number(s) can be found below: https://zenodo.org/records/16949744.
