# Supplementary material for: The veridical Near-Death Experience Scale: construction and a first validation with human and artificial raters
Source: Front Psychol. 2025 Oct 16;16:1661390. doi: 10.3389/fpsyg.2025.1661390 (PMC12573288; doi:10.3389/fpsyg.2025.1661390)
Supplement: Supplementary file 1 [file Supplementary_file_1.docx]

**Version 06/06/2025**

**Veridical Near-Death Experiences Scale (vNDEScale)**

**by**

**Bruce Greyson^[[1]](#footnote-2)^, Jeffrey Long, Janice Holden, Jean-Pierre Jourdan, Robert A. King, Robert Mays, Suzanne Mays, Pasricha Satwant, Titus Rivas, Natasha Tassell-Matamua, Pim van Lommel, Marjorie Woollacott and Patrizio Tressoldi**

This scale is intended for those who wish to assess the evidential strength of perceptions during near-death experiences (NDEs) following a set of criteria reflecting the timing, physiological state of the experiencer, third-person verification, and their characteristics.

Scale Direction Consistency:

All scales are ranked from 1 (lowest evidential strength) to 4 (highest evidential strength).

If there is no information related to a specific criterion, rate it as 1 except for criterion 7.

In case of multiple NDEs, rate each experience separately.

Reference or source (e.g. weblink) of the NDE to be assessed: _______________________________

_________________________________________________________________________________

Rater’s identity: ____________________________________________________________________

**Criteria:**

**1. Timing of investigation:** The period of time between when the NDE occurred and when investigation of veridical aspects of the NDE was initiated independently from the time of its publication in scientific or not scientific outlets.

Rate this criterion on this scale:

| 1 – after more than 5 years following the NDE | 2 – from 1 to 5 years  following the NDE | 3 – from 3 to 12 months  following the NDE | 4 – less than three months following the NDE |
| --- | --- | --- | --- |

**Rating explanation (e.g., approximate dates of the NDE occurrence and of the investigation):**

**2. Physical state of non-responsivity (unconsciousness):** The timing of the perception(s), indicating whether they occurred during physical non-responsivity, reported by the medical staff.

Rate this criterion on this scale:

| 1 – during normal consciousness | 2 – during diminished or altered consciousness | 3 – during apparent (inferred or reported verbally by the clinicians to the investigator) unconsciousness | 4 – during documented (verified by written clinical documents) unconsciousness |
| --- | --- | --- | --- |

**Rating explanation (e.g. description of the state of consciousness):**

**3. Cardiac, or respiratory arrest or cessation of brain activity:** The timing of the perception(s), indicating whether they occurred during cardiac or respiratory arrest or cessation of brain function, reported by the medical staff.

Rate this criterion on this scale:

| 1 – normal cardiac and respiratory function | 2 – abnormal cardiac, respiratory, or brain activity | 3 – apparent (inferred or reported verbally by the clinicians to the investigator) cardiac or respiratory arrest or cessation of brain activity | 4 – documented (verified by written clinical documents) cardiac or respiratory arrest or cessation of brain activity |
| --- | --- | --- | --- |

**Rating explanation (e.g., evidence of cardiac or respiratory arrest):**

**4. Third-person verification:** Accuracy of the perceptions verified by at least one credible source besides the near-death experiencer, such as medical personnel or other trustworthy witness, documented through such sources as published testimony, interview, and/or medical records.

Rate this criterion on this scale:

| 1 – no third-person verification | 2 – undocumented third-person verification | 3 – documented (e.g. available interview by a credible source) verification from one third-person source | 4 – documented (e.g. available interview by a credible source) verification from more than one third-person source |
| --- | --- | --- | --- |

**Rating explanation (e.g., evidence or identity of third-person verification):**

**5. Possible physical explanation:** The nature of the perception was such that it could be accounted for through physical sensory cues or logical inference.

Rate this criterion on this scale:

| 1 – perceptions that might be accounted for by sensory cues | 2 – perception that cannot be accounted by sensory cues, but might be accounted for by logical inference | 3 – unlikely that perceptions could have conventional physical explanation | 4 – extremely unlikely for perceptions to have conventional physical explanation |
| --- | --- | --- | --- |

**Rating explanation (e.g., source of perceived events, possibility of sensory cues):**

**6. Number of verified perceptions:** Number of verified persons, objects, environment characteristics or events perceived in the physical environment, whether immediate (e.g. hospital) or remote (e.g. in a distant city or house), during the period(s) of physical non-responsivity. For example, if a near-death experiencer reports: “*I heard the monitor go from beeping to a steady alarm. Dr. A bumped a cart to his left, forcing it to bump hard into Nurse B’s right hip. Dr. A said, “Sorry.*”, the number of verified items is four: (1) “*I heard the monitor go from beeping to a steady alarm*”; (2) “*Dr. A bumped a cart to his left*”; (3) “ *forcing it to bump hard into Nurse B’s right hip*”; (4) “*Dr. A said, “Sorry.*”

Rate this criterion on this scale:

| 1 – one | 2 – 2 or 3 | 3 – 4 to 7 | 4 – more than 7 |
| --- | --- | --- | --- |

**Rating explanation (e.g., examples of perceived veridical perceptions):**

**7. Erroneous perceptions:** Number of persons, objects, environment characteristics or events that the experiencer reported having perceived during the NDE yet were later found to be inaccurate perceptions. If the score on criterion 4 (Third-person verification**)** is 1 or 2, rate this criterion as 1.

Rate this criterion on this scale:

| 1 – all erroneous perceptions | 2 – less than 50% of correct perceptions | 3 – more than 50% of correct perceptions | 4 – no erroneous perceptions |
| --- | --- | --- | --- |

**Rating explanation** (e.g. examples of erroneous perceptions).

**8. Clarity of verified perceptions**: Clarity of the experiencer’s reported verified perceptions and the memory of them.

Rate this criterion on this scale:

| 1 – unclear or vague | 2 – less clear or precise than normal physical perceptions | 3 – as clear and precise as normal physical perceptions | 4 –clearer and more precise than most normal physical perceptions |
| --- | --- | --- | --- |

**Rating explanation (e.g., details of the veridical perceptions):**

**Other** **Comments** (explain).

**Total score:**

Total score will range from 8 to 32.

Level of evidential strength:

First quartile (8-14): Very Low evidential strength

Second quartile (15-20): Low evidential strength

Third quartile (21-26): Moderate evidential strength

Fourth quartile (27-32): Strong evidential strength, with score of 3 or 4 on criterion 4.

1. Corresponding authors: Bruce Greyson, email: [CBG4D@uvahealth.org](mailto:CBG4D@uvahealth.org) ; Patrizio Tressoldi, email: [patrizio.tressoldi@unipd.it](mailto:patrizio.tressoldi@unipd.it) [↑](#footnote-ref-2)
